# Use of Actigraphy on sleep outcomes for dementia family caregivers: an integrative review

**DOI:** 10.1093/geroni/igab046.2946

**Published:** 2021-12-17

**Authors:** Eunae Ju, Melissa Pinto, Jung-Ah Lee

**Affiliations:** 1 University of California Irvine, Irvine, California, United States; 2 University of California, Irvine, Irvine, California, United States

## Abstract

Sleep difficulties are one of the foremost health problems that affect family caregivers of dementia patients increasing their risk for a host of mental health problems and hastening dementia patients’ transitions to long-term care facilities. This integrative review aims to describe the objective measurement of sleep quality parameters of family caregivers using actigraphy and how well they are associated with self-reported subjective measures of sleep outcomes and psychological states. A search was performed using PubMed, CINAHL, and PsycInfo including articles from 2011 to 2020. Twenty studies met the inclusion criteria. Five sleep interventions (2 RCTs and 3 pre-post design) were found, including multi-component interventions (e.g., sleep hygiene, walking, day-time light therapy) that used actigraphy and other self-report measures. Duration of wearing actigraphy (wrist band/watch) varied in studies (3-days to 8-weeks). Most studies reported high accuracy and sensitivity of actigraphy. Sleep parameters measured by actigraphy included ‘total sleep time’, ‘sleep efficiency’, ‘deep/light sleep’, or ‘wake time after sleep onset’. In eight studies, sleep parameters measured by actigraphy were significantly associated with sleep outcomes measured by sleep related self-reported scales (Epworth Sleepiness Scale, Pittsburgh Sleep Quality Index, all Ps<.05). Eleven studies used actigraphy to examine sleep measures associated with various mental health states (depression, burden, stress, positive/negative affect) and found significant relationships (All Ps <.05). Findings support that use of actigraphy for dementia family caregivers is a valid measure of sleep parameters when compared with their sleep self-reports. Furthermore, it was found that actigraphy sleep measures were significantly associated with psychological outcomes.

